# COVID-19 prevalence estimation: Four most affected African countries

**DOI:** 10.1016/j.idm.2020.10.002

**Published:** 2020-10-12

**Authors:** Adewale F. Lukman, Rauf I. Rauf, Oluwakemi Abiodun, Olajumoke Oludoun, Kayode Ayinde, Roseline O. Ogundokun

**Affiliations:** a Department of Mathematics and Computer Science, Landmark University, Omu-Aran, Kwara State, Nigeria; b Department of Statistics, University of Abuja, Abuja, Nigeria; c Department of Statistics, Federal University of Technology, Akure, Nigeria

**Keywords:** COVID-19, Prevalence, Statistical tools, Time series model, ARIMA models, Forecasting, Exponential growth

## Abstract

The world at large has been confronted with several disease outbreak which has posed and still posing a serious menace to public health globally. Recently, COVID-19 a new kind of coronavirus emerge from Wuhan city in China and was declared a pandemic by the World Health Organization. There has been a reported case of about 8622985 with global death of 457,355 as of 15.05 GMT, June 19, 2020. South-Africa, Egypt, Nigeria and Ghana are the most affected African countries with this outbreak. Thus, there is a need to monitor and predict COVID-19 prevalence in this region for effective control and management. Different statistical tools and time series model such as the linear regression model and autoregressive integrated moving average (ARIMA) models have been applied for disease prevalence/incidence prediction in different diseases outbreak. However, in this study, we adopted the ARIMA model to forecast the trend of COVID-19 prevalence in the aforementioned African countries. The datasets examined in this analysis spanned from February 21, 2020, to June 16, 2020, and was extracted from the World Health Organization website. ARIMA models with minimum Akaike information criterion correction (AICc) and statistically significant parameters were selected as the best models. Accordingly, the ARIMA (0,2,3), ARIMA (0,1,1), ARIMA (3,1,0) and ARIMA (0,1,2) models were chosen as the best models for SA, Nigeria, and Ghana and Egypt, respectively. Forecasting was made based on the best models. It is noteworthy to claim that the ARIMA models are appropriate for predicting the prevalence of COVID-19. We noticed a form of exponential growth in the trend of this virus in Africa in the days to come. Thus, the government and health authorities should pay attention to the pattern of COVID-19 in Africa. Necessary plans and precautions should be put in place to curb this pandemic in Africa.

## 1 Introduction

Infectious diseases are becoming prominent and have continued to infect and reduce human populations. The world at large has been confronted with several disease outbreak which has posed and still posing a serious menace to public health globally. These include the severe acute respiratory syndrome (SARS) which resulted in eight hundred (800) deaths out of about approximately eight thousand (8000) cases in 2002, the H1N1 prevalent that recorded about eighteen thousand five hundred (18500) deaths in 2009. The Middle East respiratory syndrome (MERS) outbreak which led to the demise of eight hundred (800) people out of two thousand five hundred (2500) cases in the year 2012. The Ebola epidemic, with eleven thousand three hundred and ten (11310) deaths out of twenty-eight thousand six hundred and sixteen (28616) cases in 2014 (Anjorin, 2020). Recently, there begins another pandemic called COVID-19. COVID-19 is a novel kind of coronavirus had been recognized from a family of zoonotic coronaviruses. It includes the severe acute respiratory syndrome coronavirus (SARS-CoV) and the Middle East Respiratory Syndrome Coronavirus (MERS-CoV) (Ogundokun et al., 2020; Wang et al., 2020). Coronavirus disease (COVID-19) outbreak with global deaths of 457,355 out 8,622,985 positive cases identified as of 15.05 GMT, June 19, 2020 (WHO, 2020).

COVID-19 is a respiratory infectious disease caused by a new strain of coronavirus that causes illness in humans. Scientists are still learning about the disease, and think that the virus began in animals. At some point, one or more humans acquired infection from an animal, and those infected humans began transmitting the infection to other humans. The disease spreads from person to person through infected air droplets that are projected during sneezing or coughing. It can also be transmitted when humans have contact with hands or surfaces that contain the virus and touch their eyes, nose, or mouth with the contaminated hands (Ayinde et al., 2020). COVID-19 was first reported in China, but it has now spread throughout the world. Before COVID-19 was officially declared as a pandemic by the world health organization on March 11, 2020, the virus has escalated and covered a large area of more than 114 countries and territories.

In Africa, as of June 16, 2020, 251866 cases were recorded with 6769 deaths, and 114308 Recoveries have been reported in fifty-five (55) African countries which are about 3.2% of all incidents reported globally (Africa CDC, 2020). Five of these African countries account for 63% of these cases. These include South Africa (34%), Egypt (16%), Nigeria (6%), Ghana (4%), and Cameroon (3%).

In Nigeria the first COVID-19 case was reported on February 28, 2020, as of 10:40 a.m. CEST, June 16, 2020, there have been 16,658 confirmed cases of COVID-19 with 10,885 active cases, 424 deaths and 5349 discharged, (NCDC, 2020). In South Africa, the first case of COVID-19, was reported on March 5, 2020, and as of 10:40 a.m. CEST, June 16, 2020, there have been 1,148,933 Tests Conducted, 73,533 positive cases identified/confirmed cases, 39,867 Recoveries of COVID-19 with 1568 deaths. In Egypt, the first case of COVID-19, was reported on February 14, 2020, and as of 10:40 a.m. CEST, June 16, 2020, there have been 46,289 confirmed cases of COVID-19 with 1672 deaths. In Ghana, the first case of COVID-19, was reported on March 14, 2020, and as of 10:40 a.m. CEST, June 16, 2020, there have been 11,964 confirmed cases of COVID-19 with 54 deaths.

Recently, Ayinde et al. (2020) applied the linear regression model and some other curve estimation model to predict the prevalence of COVID-19 in Nigeria. SEIR model and Regression model have been used for predictions for COVID 19 in India (Pandey et al., 2020). Linear Regression Analysis was adopted to predict the number of deaths in India due to SARS-CoV-2 (Ghosal et al., 2020). Among the various statistical tools used to predict epidemic cases is the AutoRegressive Integrated Moving Average (ARIMA) model. A high number of researchers have applied the ARIMA model for disease prevalence/incidence prediction in different diseases outbreak. These include Guan et al. (2004), Earnest et al. (2005), Gaudart et al. (2009), Liu et al. (2011), Nsoesie et al. (2013), Zheng et al. (2015), He and Tao (2018), Fang et al. (2020), Polwiang (2020) and Cao et al. (2020). Sato (2013) presented the process of using the ARIMA model in evaluating infectious and non-infectious disease management. ARIMA model has been used to ameliorate the forecast accuracy of several epidemic diseases (Pan et al., 2016). (Perone, 2020) estimated an autoregressive integrated moving average (ARIMA) model to forecast the epidemic by using the Italian epidemiological data at the national and regional level. An Auto-Regressive Integrated Moving Average (ARIMA) model prediction was performed on the Johns Hopkins epidemiological data to predict the epidemiological trend of the prevalence and incidence of COVID-2019 (Benvenuto et al., 2020). Rauf and Oladipo (2020) forecasted the spread of COVID-19 in Nigeria using the ARIMA model. Auto-Regressive Integrated Moving Average (ARIMA) model was used to predict the pattern of confirmed cases of COVID-19 in different countries (Tania et al., 2020). Hitesh et al. (2020) developed an ARIMA model and then employed it for forecasting future COVID-19 cases in India. Ceylan (2020) applied the auto-regressive integrated moving average (ARIMA) model to predict the prevalence of COVID-19 in Italy, Spain, and France.

In this study, we will focus on the estimation and prediction of COVID-19 prevalence in the following countries that are Africa epicentre: South Africa, Egypt, Nigeria, and Ghana. The datasets examined in this analysis spanned February 21, 2020 to June 16, 2020. According to Cao et al. (2020), ARIMA models have made significant progress in the health sciences as well as different fields for an efficient epidemic forecast. This simple time series model was adopted in this study. Finally, this study will help to monitor the trends of this pandemic in Africa, provide a reliable estimate and forecast. It will further help the federal government to take decisions to curb the outbreak.

## 2 Methodology

The COVID-19 dataset employed in this study was taken from the WHO website (https://www.who.int/emergencies/diseases/novel-coronavirus-2019/situation-reports/), and the analysis was done using Gretl software. The descriptive statistics of the COVID-19 data of the Africa COVID-19 epicentres between 28/02/2020–15/06/2020 are given in Table 1. According to Box et al. (2015), at least 30 observations are required for stable and effective ARIMA modelling. The sample size in this study is more than 30. We predicted COVID-19 prevalence of South Africa, Egypt, Nigeria and Ghana over the next twenty days with 95% relative confidence intervals.

**Table 1:** Descriptive statistics on COVID-19 daily confirmed cases in the South-Africa, Egypt, Nigeria and Ghana.

Countries	Minimum	Maximum	Sum	Mean	Std. Deviation	Skewness	Kurtosis
	Statistic	Statistic	Statistic	Statistic	Statistic	Statistic	Statistic
SA	0	4302	70038	686.65	950.29	1.829	3.012
Nigeria	0	681	16085	147.57	167.447	1.122	.750
Ghana	0	921	11964	125.94	175.94	1.966	4.896
Egypt	0	1676	44597	437.22	498.01	1.153	.062

Table 1 shows that among the epicentres, the highest confirmed case of COVID-19 is from South-Africa as of June 15, 2020. There are days each of those countries do not record any instances of COVID-19. An average of 687, 437, 148 and 126 people are reported to have COVID-19 in South-Africa, Egypt, Nigeria and Ghana daily respectively.

**Fig. 1:**
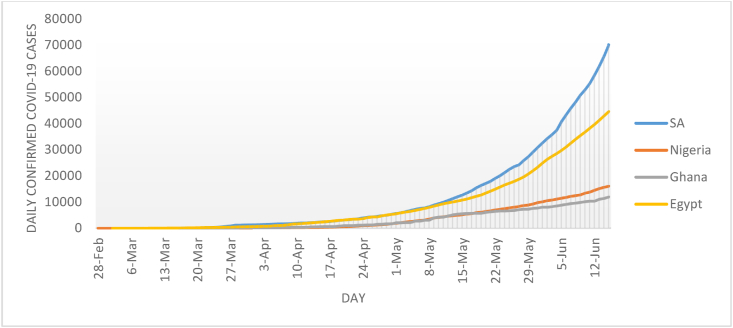
Daily confirmed COVID-19 cases.

The skewness values for each of the countries are higher than one (1), which shows that the daily cases reported in each country are highly skewed to the right. The result of the Kurtosis further indicates the dataset is not symmetric. Among these countries, Ghana has the least number of reported cases daily. It is very clear from Fig. 1 that Nigeria was the first among these countries to publish the first COVID-19 cases on February 28, 2020. Both South-Africa and Egypt recorded their first cases on March 6, 2020, while Ghana first case happened four days after.

**Fig. 2:**
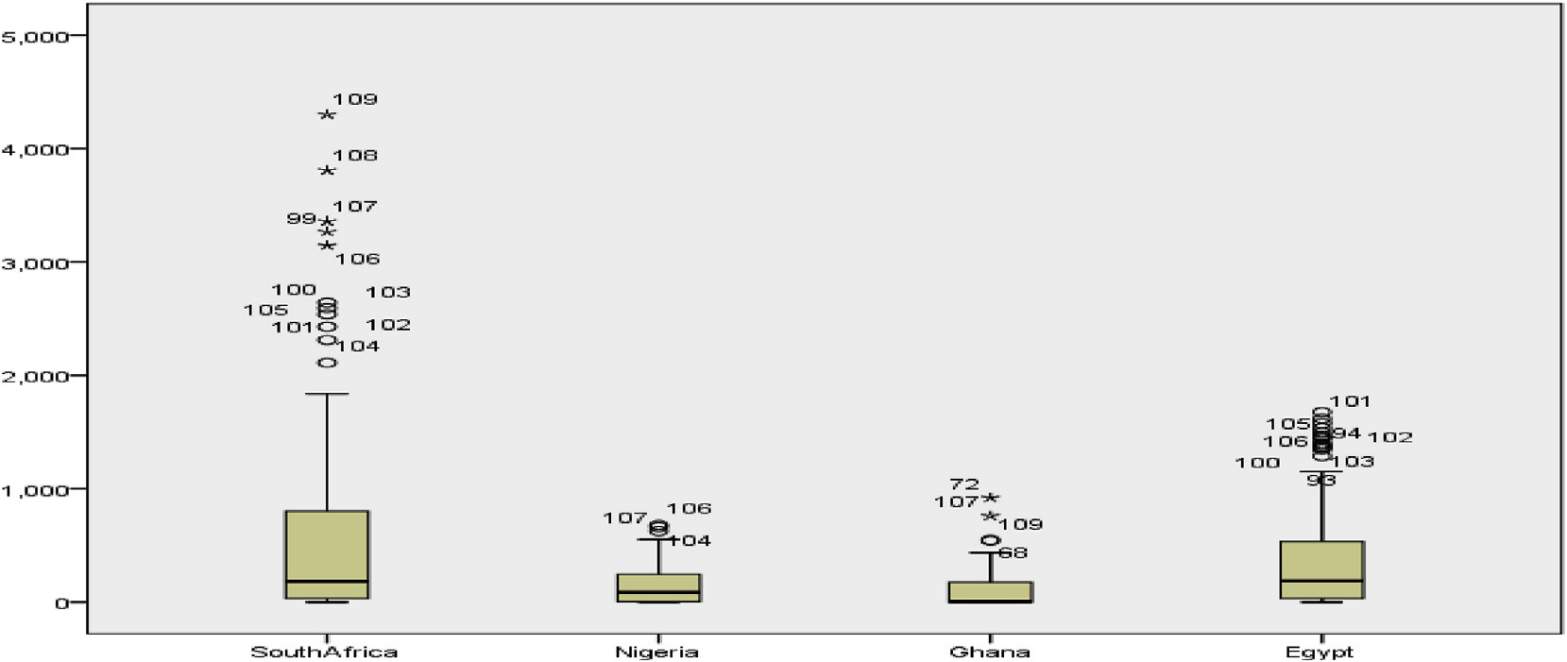
Box plot of the daily number of cases reported in the four countries.

**Fig. 3:**
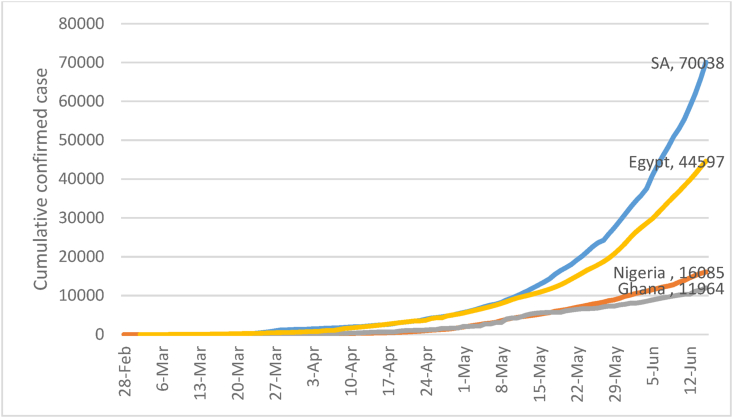
Cumulative confirmed COVID-19 cases.

Also, from Fig. 1, we observed a slow movement in the progression of the number of COVID-19 cases reported daily in the four countries until March 23. The number of cases reported daily until March 24 were in their tens. However, as seen in Fig. 1, South-Africa began to experience a peak by recording hundreds of COVID-19 cases daily. Fig. 1 confirm that the overall prevalence of COVID-19 used in this study does not show seasonal patterns. The box-plot in Fig. 2 provides a good insight into the number of outlying cases. For instance, COVID-19 cases in Nigeria were outlying in the following days: 104, 106 and 107. About eleven (11) outlying cases were identified in South-Africa. Four and eight outlying points were detected in Ghana and South-Africa, respectively. Fig. 3 is the time series plot of the cumulative confirmed cases of COVID-19.

### 2.1 ARIMA models

According to Fanoodi et al. (2019), time-series data are a set of observations indexed and ordered in time. Time series analysis seeks to analyse time-series data to reveal the characteristics of the data and obtained meaningful. Time series forecasting employed time series model to predict future values of the series based on previously observed values (Liu et al., 2011; Elevli et al., 2016; He and Tao, 2018; Benvenuto et al., 2020, Ceylan, 2020). The autoregressive integrated moving average (ARIMA) models is a popularly used time series model introduced by Box and Jenkins in the 1970s (Box et al., 2015). The ARIMA models take into account the non-stationarity aspect of the data with the possibility of one or two differencing. The model is applicable for all kinds of data, including trend, seasonality, and cyclicity (Ceylan, 2020). It is generally denoted ARIMA (p,d,q) where p is the order of autoregressive model, d is the degree of differencing, and q is the order of moving average (Li et al., 2019). The ARIMA model is a generalization of the autoregressive moving average (ARMA) model. The autoregressive (AR) involves regressing the variable of interest *Y*_*t*_ on its lagged values *Y*_*t−1*_, *Y*_*t−2*_,..,*Y*_*t−p*_. The moving average (MA) part involves regressing the series *Y*_*t*_ on the current residuals $\varepsilon_{t}$ and its lagged residual series $\varepsilon_{t-1,}\varepsilon_{t-2},\varepsilon_{t-3,.}...,\varepsilon_{t-q}.$ The integrated (I) denotes taking the difference between the actual time series data and its lagged values. The differencing can be done once or twice. The ARMA(*p*,*q*) model consist of the AR and MA process and it is defined as follows:

(1) $$Y_{t}=\alpha +\phi_{1}Y_{t-1}+\phi_{2}Y_{t-2}+...+\phi_{p}Y_{t-p}+\varepsilon_{t}-\theta_{1}\varepsilon_{t-1}-\theta_{2}\varepsilon_{t-2}-...-\theta_{q}\varepsilon_{t-q}.$$

where α is a constant, ϕ and θ are the autoregressive and moving average parameters, respectively. $Y_{t}$ is the observed value at time t and $\varepsilon_{t}$ is the value of the residual at time t such that $\varepsilon_{t}\sim N\left(0,\sigma^{2}\right).$

The ARIMA modelling approach includes model identification and model selection, parameter estimation, diagnostic checking, and forecasting. The first steps of model identification and model selection are to ensure that the time series variable is stationary and to identify if the series is seasonal or not. The autocorrelation (ACF) and the partial autocorrelation (PACF) functions of the dependent variable was adopted for model identification and selection. The parameters in the model are estimated using maximum likelihood estimation. We conduct statistical model checking by testing whether the estimated model conforms to the specifications of a stationary univariate process. In particular, the residuals should be independent of each other and constant in mean and variance over time. Misspecification can be identified by plotting the mean and variance of residuals over time and performing a Ljung–Box test. Alternatively, we can plot the autocorrelation and partial autocorrelation of the residuals. If the estimation is inadequate, we have to return to step one and attempt to build a better model.

Augmented Dickey-Fuller test was adopted to check the stationarity status of the series (daily confirmed cases of COVID-19 in South Africa (SA), Nigeria, Ghana and Egypt). The result of the test is provided in Table 2. We observed that all the daily confirmed cases in the four countries are not stationary at the actual level. The time-series data become stable at the first difference for the following countries: Nigeria, Egypt and Ghana while that of South-Africa became stationary at the second difference. Thus, the series is ready for modelling in line with the Box-Jenken ARIMA modelling approach. The parameters of the ARIMA models were determined according to the ACF and PACF plots (see Fig. 4a, Fig. 4ba and b). Potential models combination were obtained from the ACF and the PACF plot for SA, Nigeria, Ghana and Egypt. The order of the model was determined according to ACF and PACF after applying the individual difference of the country’s prevalence series. The result of the different possible models is presented in Table 3. The ACF and PACF plots for the countries COVID-19 prevalence series are displayed in Fig. 4a, Fig. 4b, Fig. 4c, Fig. 4da–d.

**Table 2:** Unit root test (Stationarity test for the Covid 19 confirmed cases).

Augmented Dickey-Fuller Test				
Country	Transformation	Dickey-Fuller	Lag order	p-value
SA	Level	1.9046	4	0.99
	Difference 1	−7.1958	4	0.01∗
	Difference 2	−8.3223	4	0.01∗
NIGERIA	Level	−3.0307	4	0.1491
	Difference 1	−7.235	4	0.01∗
GHANA	Level	−2.831	4	0.2319
	Difference 1	−6.2974	4	0.01∗
EGYPT	Level	−0.91781	4	0.9474
	Difference 1	−7.4097	4	0.01∗

∗ significant and thus stationary at that level.

**Fig. 4a:**
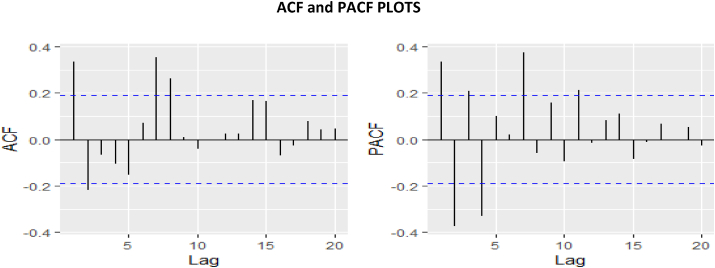
ACF and PACF for SA

**Fig. 4b:**
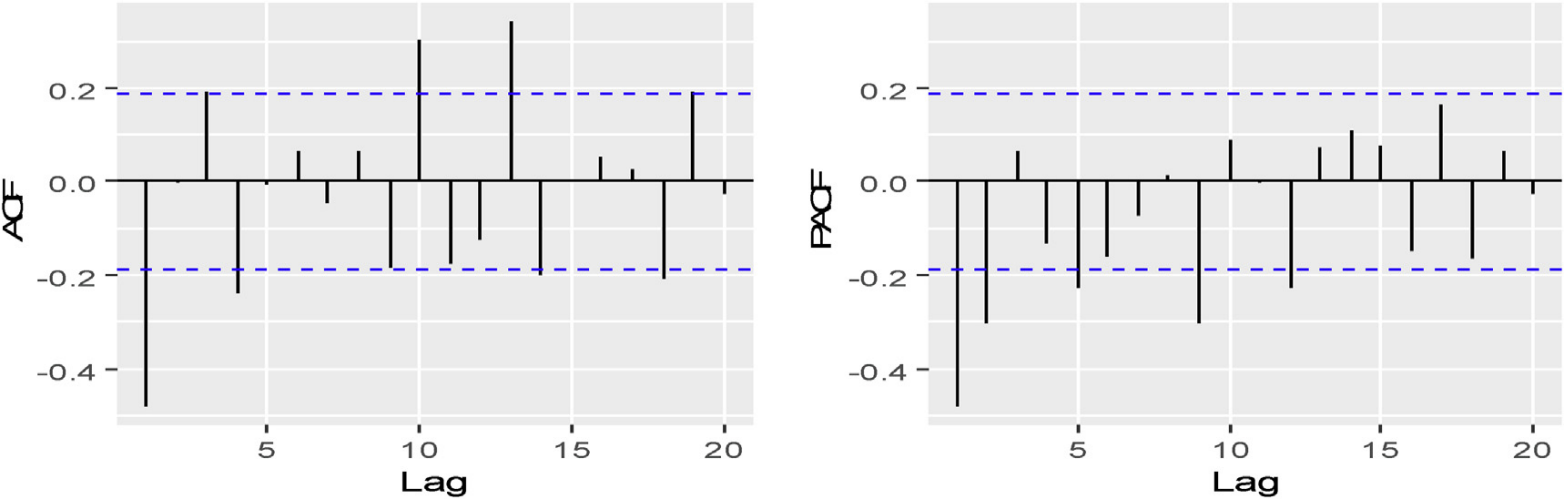
ACF and PACF for Nigeria.

**Table 3:** Comparison of tested ARIMA models.

Comparison of tested ARIMA models			
Country	Candidate Models	Selection Criterion	Best Model
		AICc	
SA	ARIMA (2,2,2)	Inf	ARIMA (0,2,3)
	ARIMA (0,2,0)	1573.273	
	ARIMA (1,2,0)	1534.298	
	ARIMA (0,2,1)	1488.462	
	ARIMA (1,2,1)	1485.743	
	ARIMA (2,2,1)	1486.195	
	ARIMA (1,2,2)	Inf	
	ARIMA (0,2,2)	1483.683	
	ARIMA (0,2,3)	1472.132	
	ARIMA (1,2,3)	Inf	
	ARIMA (0,2,4)	Inf	
	ARIMA (1,2,4)	Inf	
Nigeria	ARIMA (0,1,0) with drift	1267.746	ARIMA (0,1,1) with drift
	ARIMA (1,1,0) with drift	1241.469	
	ARIMA (0,1,1) with drift	1225.898	
	ARIMA (0,1,0)	1265.881	
	ARIMA (1,1,1) with drift	1227.949	
	ARIMA (0,1,2) with drift	1227.966	
	ARIMA (1,1,2) with drift	1229.924	
	ARIMA (0,1,1)	1229.016	
Ghana	ARIMA (0,1,0) with drift	1464.448	ARIMA (3,1,0)
	ARIMA (1,1,0) with drift	1412.372	
	ARIMA (0,1,1) with drift	Inf	
	ARIMA (0,1,0)	1462.434	
	ARIMA (2,1,0) with drift	1405.717	
	ARIMA (3,1,0) with drift	1375.752	
	ARIMA (4,1,0) with drift	1376.361	
	ARIMA (3,1,1) with drift	1376.272	
	ARIMA (2,1,1) with drift	Inf	
	ARIMA (4,1,1) with drift	Inf	
	ARIMA (3,1,0)	1374.509	
	ARIMA (2,1,0)	1403.947	
	ARIMA (4,1,0)	1375.253	
	ARIMA (3,1,1)	1375.247	
	ARIMA (2,1,1)	1384.69	
	ARIMA (4,1,1)	1376.465	
Egypt	ARIMA (0,1,0) with drift	1304.969	ARIMA (0,1,2) with drift
	ARIMA (1,1,0) with drift	1306.215	
	ARIMA (0,1,1) with drift	1305.048	
	ARIMA (0,1,0)	1305.297	
	ARIMA (1,1,2) with drift	1298.57	
	ARIMA (0,1,2) with drift	1296.403	
	ARIMA (0,1,3) with drift	1298.57	
	ARIMA (1,1,1) with drift	1302.234	
	ARIMA (1,1,3) with drift	1300.813	
	ARIMA (0,1,2)	1300.818	
	Best model: Candidate model with least AICc value

**Fig. 4c:**
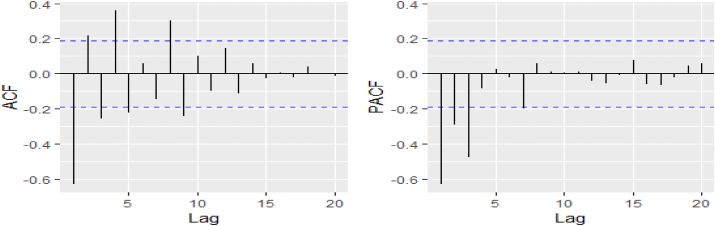
ACF and PACF for Ghana.

**Fig. 4d:**
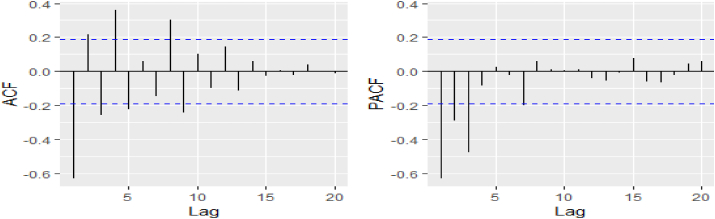
ACF and PACF for Egypt.

ARIMA models with minimum Akaike information criterion correction (AICc)and statistically significant parameters were selected as the best models. Accordingly, the ARIMA (0,2,3), ARIMA (0,1,1), ARIMA (3,1,0) and ARIMA (0,1,2) models were chosen as the best models for SA, Nigeria, and Ghana and Egypt, respectively. The models fitted the COVID-19 data reasonably well (Table 4) with a minimum AICc = 1471.74, 1225.67, 1374.12 and 1296.01 for SA, Nigeria, and Ghana and Egypt, respectively.

**Table 4:** Parameters of the Best ARIMA models.

Country	Best Model	Coefficients		s.e.	AIC	AICc	BIC	Likelihood
SA	ARIMA (0,2,3)	ma1	−1.5122	0.0875	1471.74	1472.13	1482.43	−731.87
		ma2	0.2097	0.1402				
		ma3	0.3593	0.0791				
Nigeria	ARIMA (0,1,1) with drift	ma1	−0.7444	0.0707	1225.67	1225.9	1233.71	−609.83
		Drift	4.7413	1.7271				
Ghana	ARIMA (3,1,0)	ar1	−0.9429	0.0822	1374.12	1374.51	1384.85	−683.06
		ar2	−0.6729	0.1028				
		ar3	−0.5303	0.0862				
Egypt	ARIMA (0,1,2) with drift	ma1	−0.109	0.0896	1296.01	1296.4	1306.74	−644.01
		ma2	−0.3312	0.0866				
		drift	14.978	5.1267				

The best model are estimated in Table 4 and eventually employed for forecasting the daily spread series of COVID19. The forecast is available in Table 6.

**Table 5:** Residual test of the models.

Box-Ljung test			
Country	$\chi^{2}$	df	p-value
SA	42.911	20	0.21
Nigeria	32.505	20	0.3821
Ghana	12.233	20	0.9078
Egypt	21.819	20	0.3504

**Table 6:** Prediction of total confirmed cases of COVID-19 for the next ten days according to ARIMA models with 95% confidence interval.

Date	SA			Nigeria			Ghana				Egypt		
	ARIMA (0,2,3)			ARIMA (0,1,1) with drift			ARIMA (3,1,0)				ARIMA (0,1,2) with drift		
	Forecast	Lower limit	Upper limit	Forecast	Lower limit	Upper limit		Forecast	Lower limit	Upper limit	Forecast	Lower limit	Upper limit
16-Jun-20	3932.79	3496.29	4369.29	503.27	368.15	638.40		221.39	−45.00	487.78	1605.62	1418.84	1792.39
17-Jun-20	3895.49	3409.80	4381.18	508.02	368.55	647.49		605.37	338.55	872.19	1627.36	1377.20	1877.51
18-Jun-20	4105.65	3613.27	4598.04	512.76	369.08	656.44		332.83	56.26	609.41	1642.33	1371.21	1913.45
19-Jun-20	4315.81	3812.19	4819.43	517.50	369.72	665.27		501.46	221.05	781.87	1657.31	1366.73	1947.89
20-Jun-20	4525.97	4005.70	5046.25	522.24	370.48	674.00		322.22	−3.51	647.95	1672.29	1363.48	1981.10
21-Jun-20	4736.13	4193.14	5279.12	526.98	371.34	682.62		522.29	194.06	850.51	1687.27	1361.24	2013.29
22-Jun-20	4946.29	4374.17	5518.41	531.72	372.30	691.15		364.82	23.88	705.77	1702.25	1359.87	2044.62
23-Jun-20	5156.45	4548.70	5764.20	536.46	373.34	699.59		473.73	126.77	820.69	1717.22	1359.24	2075.20
24-Jun-20	5366.61	4716.84	6016.38	541.21	374.47	707.94		370.90	3.04	738.76	1732.20	1359.27	2105.14
25-Jun-20	5576.77	4878.87	6274.67	545.95	375.67	716.22		478.08	105.53	850.63	1747.18	1359.87	2134.49
26-Jun-20	5786.93	5035.14	6538.72	550.69	376.94	724.43		388.46	4.12	772.80	1762.16	1360.99	2163.33
27-Jun-20	5997.09	5186.05	6808.13	555.43	378.28	732.57		455.37	64.19	846.56	1777.14	1362.57	2191.70
28-Jun-20	6207.25	5331.96	7082.53	560.17	379.69	740.65		395.74	−8.74	800.23	1792.11	1364.57	2219.66
29-Jun-20	6417.41	5473.27	7361.55	564.91	381.16	748.67		454.47	43.92	865.01	1807.09	1366.95	2247.23
30-Jun-20	6627.57	5610.28	7644.85	569.65	382.68	756.63		403.73	−17.05	824.52	1822.07	1369.68	2274.46
1-Jul-20	6837.73	5743.31	7932.15	574.39	384.26	764.53		443.68	15.81	871.55	1837.05	1372.74	2301.36
2-Jul-20	7047.89	5872.60	8223.17	579.14	385.89	772.38		409.01	−29.05	847.07	1852.03	1376.09	2327.96
3-Jul-20	7258.05	5998.40	8517.69	583.88	387.57	780.19		441.73	−3.00	886.45	1867.00	1379.73	2354.28
4-Jul-20	7468.21	6120.90	8815.51	588.62	389.29	787.94		413.02	−40.67	866.71	1881.98	1383.62	2380.35
5-Jul-20	7678.37	6240.28	9116.45	593.36	391.06	795.65		436.46	−24.29	897.21	1896.96	1387.75	2406.17

From the histogram of forecast errors in Fig. 5a, Fig. 5b, Fig. 5c, Fig. 5da–d, for the prevalence of COVID-19 series of SA, Nigeria, Ghana and Egypt respectively, it seems that some of the forecast errors are not normally distributed with mean zero. However, adopting the formal test using the Chi-square test in Table 5 indicates that the forecast errors are normally distributed. It suggests that the ARIMA models provide an adequate predictive model of the prevalence of COVID-19 in SA, Nigeria, Ghana and Egypt. Furthermore, the assumptions upon which the prediction intervals were based are thus valid.

**Fig. 5a:**
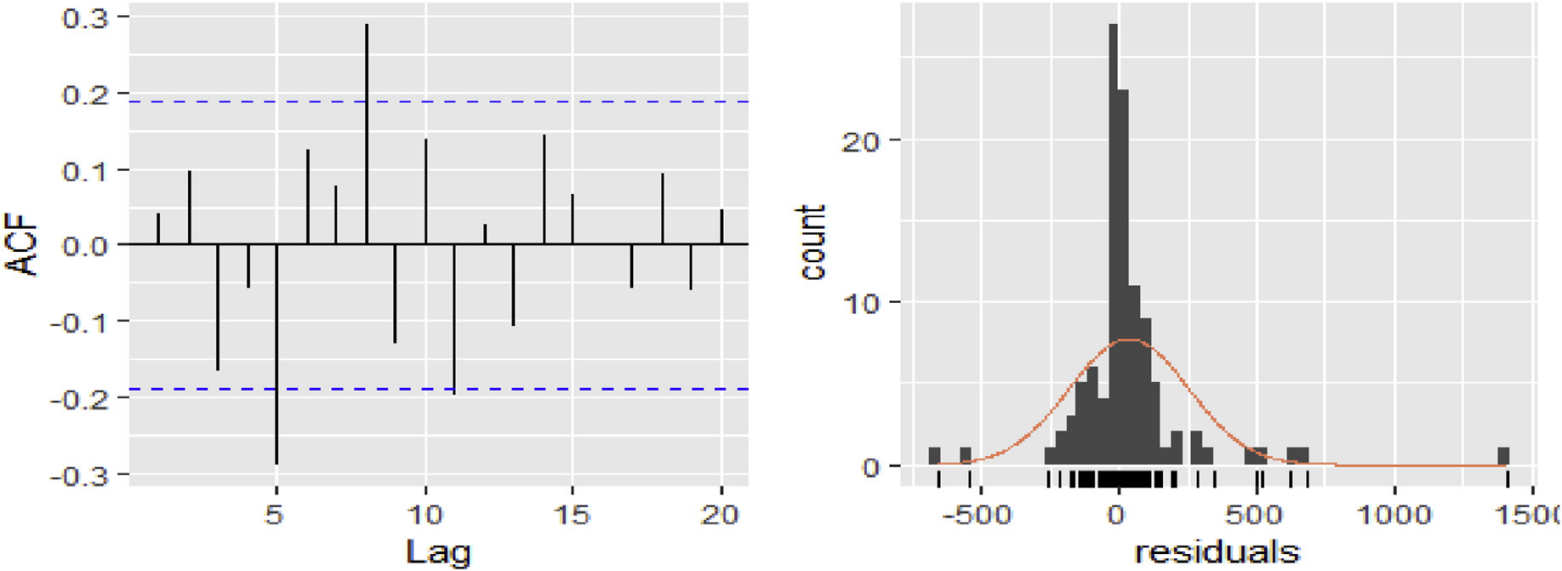
Residual from ARIMA (0,2,3) [SA].

**Fig. 5b:**
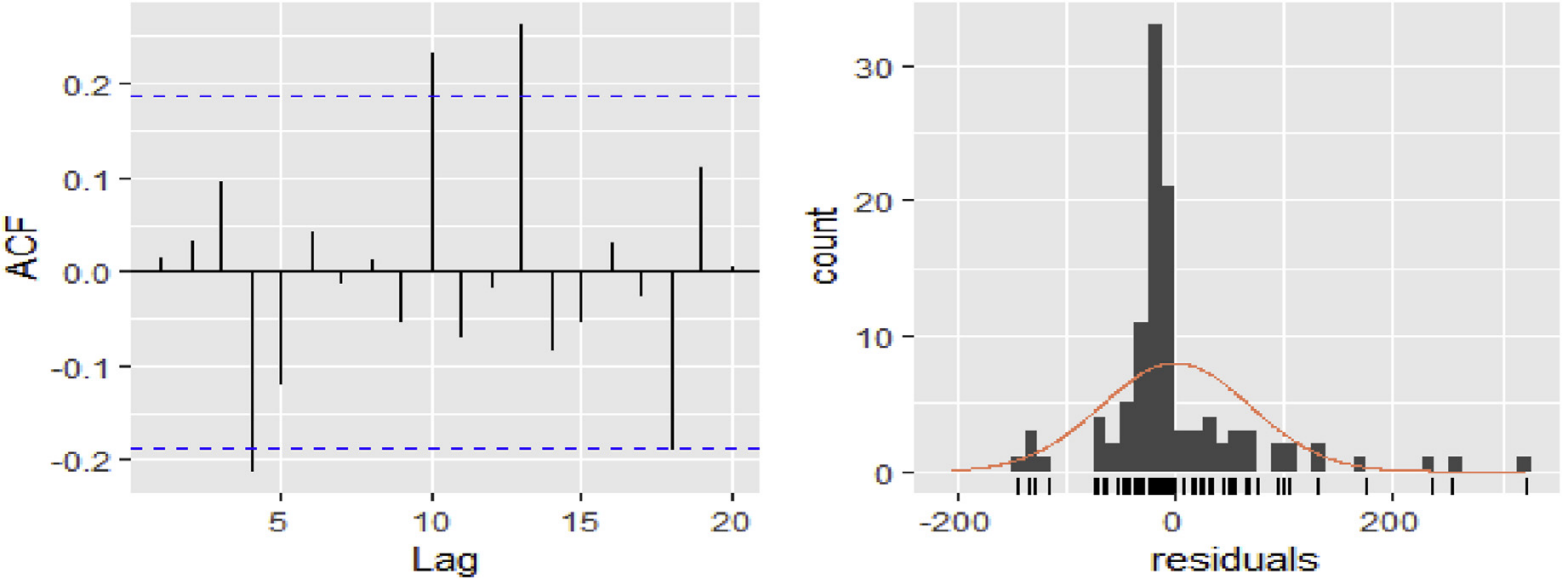
Residual from ARIMA (0,1,1) [Nigeria].

**Fig. 5c:**
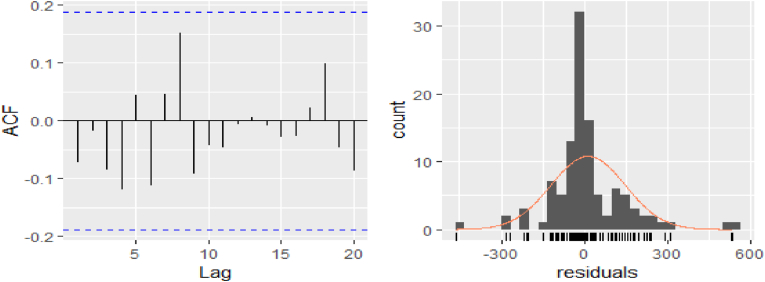
Residual from ARIMA (3,1,0) [Ghana].

**Fig. 5d:**
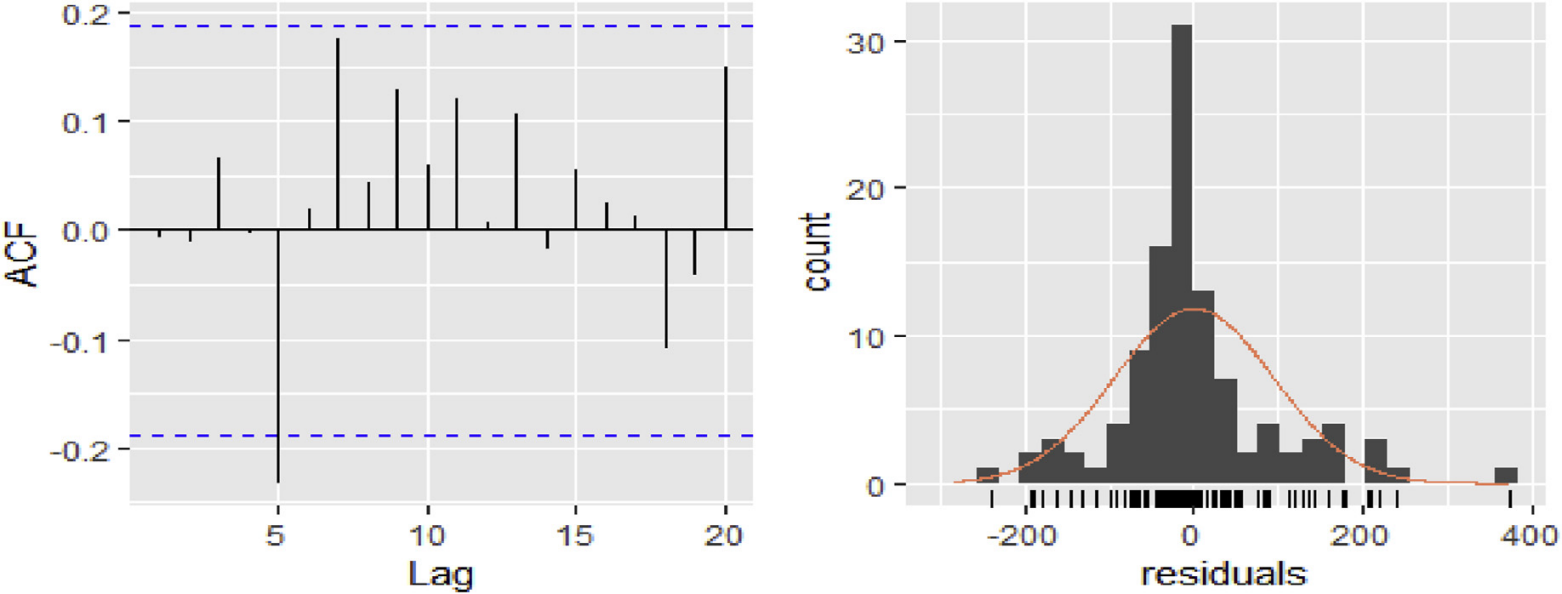
Residual from ARIMA (0,1,2) [Egypt].

As shown in Table 6, the daily spread data from June 16 to July 5, 2020 (20 days) were predicted using the ARIMA (0,2,3), ARIMA (0,1,1), ARIMA (3,1,0) and ARIMA (0,1,2) models for SA, Nigeria, and Ghana and Egypt, respectively. Based on the box-Ljung test, the results suggested that the predicted values fitted well with the actual values. The fitted and forecasted values are presented in Fig. 6a, Fig. 6b, Fig. 6c, Fig. 6da–d. We observed an exponential increase in the trends of future COVID-19 cases in the selected African countries. However, the growth rate is not sporadic in Ghana. The result from Table 6 shows that the forecasted COVID-19 prevalence for the first four most affected Africa countries as at July 3 is expected to be as follows: 59998.4-8517.69 in SA, 583.88 to 780.19 in Nigeria, −3 to 886.45 in Ghana, 1379.73–2354.28 in Egypt. Ghana is the only country that tends to experience a rapid decrease. It is also important to state here that Ghana is the only country among these countries that have recorded the least number of death of COVID-19 infected patients. For other countries, there is no downward trend movement in the predicted number of confirmed cases. The result of this study is a bit alarming, especially for South-Africa. Due to the recent relaxation in lockdown in each of these countries, there is a tendency that clinical and social problems will be unmanageable and consequently leads to crisis.

**Fig. 6a:**
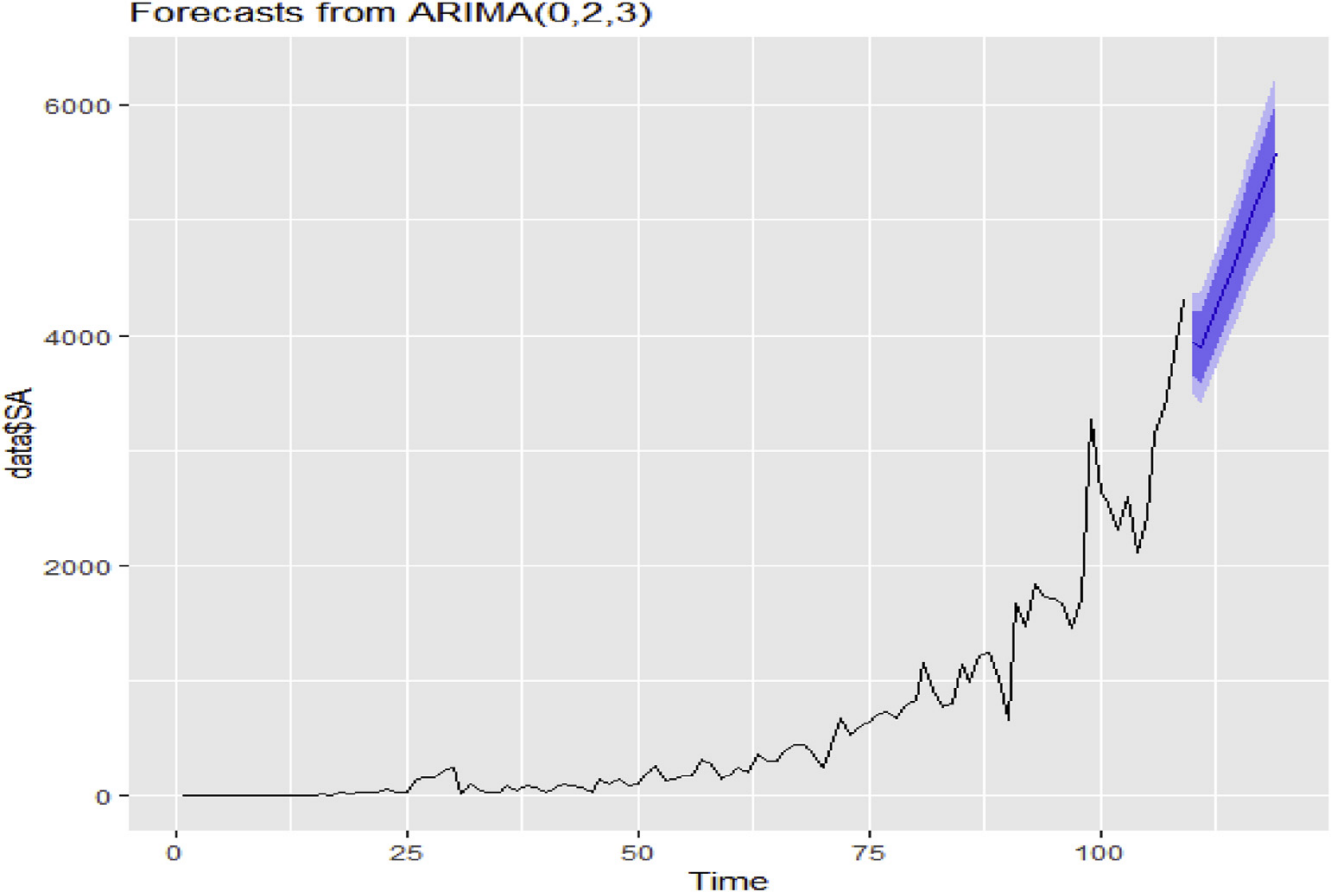
COVID-19 confirmed cases for the first one hundred and nine days and its forecast values for SA.

**Fig. 6b:**
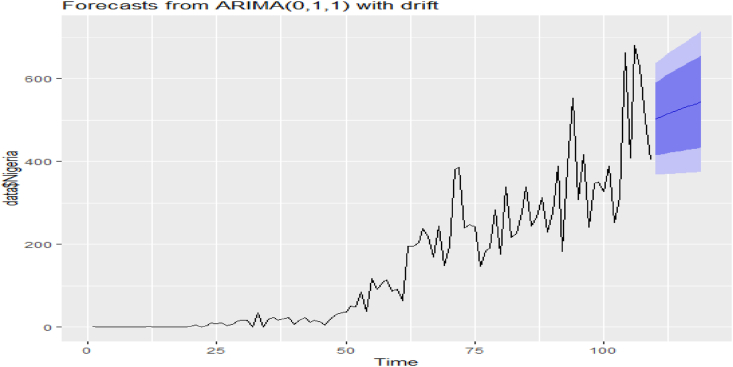
COVID-19 confirmed cases for the first one hundred and nine days and its forecast values for Nigeria.

**Fig. 6c:**
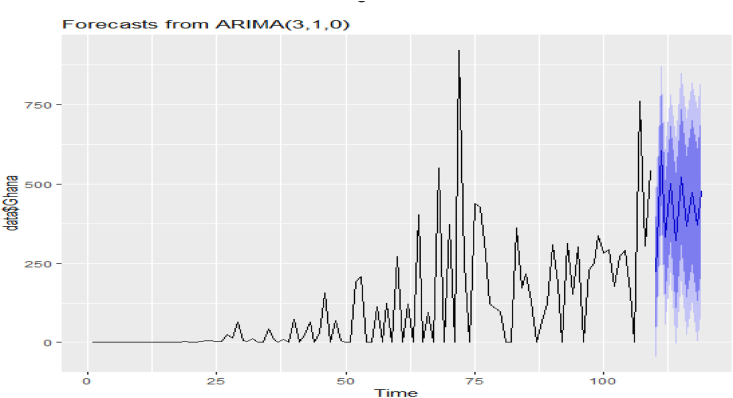
COVID-19 confirmed cases for the first one hundred and nine days and its forecast values for SA.

**Fig. 6d:**
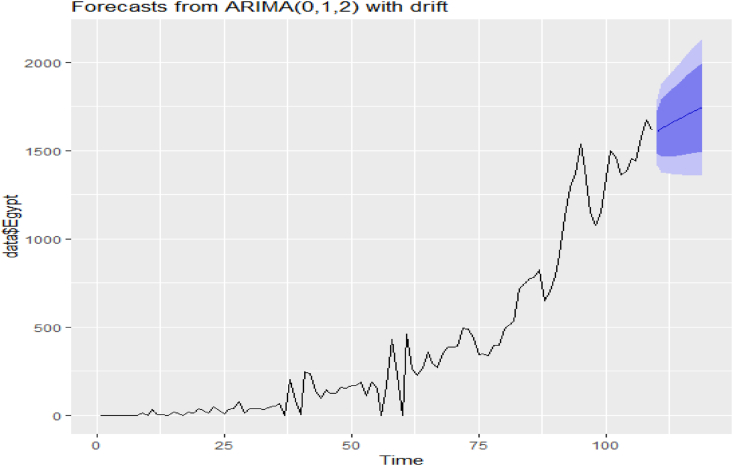
COVID-19 confirmed cases for the first one hundred and nine days and its forecast values for Egypt.

## 3 Conclusion

In this study, we presented the time series modelling for the novel COVID-19, which emerge recently in Wuhan China. The ARIMA models were applied to the daily confirmed COVID-19 cases of four African countries that are the first four most affected countries: South-Africa, Egypt, Nigeria and Ghana. We obtained the best ARIMA models for each of the countries and made a daily forecast from 16th June to July 5, 2020. The result shows that the daily number of COVID-19 cases in these epicentres will continue to increase especially in South-Africa, Nigeria and Egypt. There is a downward upward movement in Ghana. The country does not exhibit exponential progression as obtainable in other countries. The number of death rate in Ghana have also shown that the country can manage the situation well than the three others. The forecast indicates that there is a tendency of more cases, especially in the South-Africa if the outbreak is not well control. The findings in this study show that the government still have a lot to do in managing this present pandemic. We believe this study will help the government and other health authorities to plan and supply resources effectively for effective management of this pandemic in the days to come.
